# C/EBPβ regulates *Vegf* gene expression in granulosa cells undergoing luteinization during ovulation in female rats

**DOI:** 10.1038/s41598-018-36566-y

**Published:** 2019-01-24

**Authors:** Masahiro Shinagawa, Isao Tamura, Ryo Maekawa, Shun Sato, Yuichiro Shirafuta, Yumiko Mihara, Maki Okada - Matsumoto, Toshiaki Taketani, Hiromi Asada, Hiroshi Tamura, Norihiro Sugino

**Affiliations:** Department of Obstetrics and Gynecology, Yamaguchi University Graduate School of Medicine, Minamikogushi 1-1-1, Ube, 755-8505 Japan

## Abstract

The ovulatory LH-surge increases *Vegf* gene expression in granulosa cells (GCs) undergoing luteinization during ovulation. To understand the factors involved in this increase, we examined the roles of two transcription factors and epigenetic mechanisms in rat GCs. GCs were obtained from rats treated with eCG before, 4 h, 8 h, 12 h and 24 h after hCG injection. *Vegf* mRNA levels gradually increased after hCG injection and reached a peak at 12 h. To investigate the mechanism by which *Vegf* is up-regulated after hCG injection, we focused on C/EBPβ and HIF1α. Their protein expression levels were increased at 12 h. The binding activity of C/EBPβ to the *Vegf* promoter region increased after hCG injection whereas that of HIF1α did not at this time point. The C/EBPβ binding site had transcriptional activities whereas the HIF1α binding sites did not have transcriptional activities under cAMP stimulation. The levels of H3K9me3 and H3K27me3, which are transcriptional repression markers, decreased in the C/EBPβ binding region after hCG injection. The chromatin structure of this region becomes looser after hCG injection. These results show that C/EBPβ regulates *Vegf* gene expression with changes in histone modifications and chromatin structure of the promoter region in GCs undergoing luteinization during ovulation.

## Introduction

Angiogenesis is required for the corpus luteum (CL) formation^1,2^. After the surge of luteinizing hormone (LH), endothelial cells in the theca cell layer proliferate and start to invade the granulosa cell layer during ovulation. This is the first process to form the CL^3^. Endothelial cells rapidly form blood vessels and vascular network in the CL, which makes CL highly vascularized organs. This event is necessary for CL continuing to produce progesterone for establishing and maintaining pregnancy. In previous studies, angiogenic factors that are responsible for luteal development have been identified^4^. Vascular endothelial growth factor (VEGF) is one of them and is highly expressed in rat and human CL^5^. VEGF in the ovary is involved in physiologic development, maintenance and maturation of blood vessels and contributes to rapid vascularization during CL formation. Therefore, VEGF is one of important genes, which leads to CL development^6,7^. The LH surge changes a number of gene expressions in granulosa cells (GCs) during ovulation^8^. Although VEGF gene expression is increased during ovulation, the detailed molecular mechanism of this regulation has been unclear. It is well known that hypoxia inducible factor 1-alpha (HIF1α) induced by hypoxic conditions increases VEGF expression in many types of cells^9–11^. However, it is unclear whether HIF1α also regulates VEGF expression in GCs undergoing luteinization.

CCAAT/enhancer-binding protein beta (C/EBPβ) is a transcription factor involved in follicle rupture and following CL formation^12,13^. In C/EBPα and β knockout mice, the expressions of angiogenesis-related genes are suppressed and vascular development in CL is defective^12^. These findings led us to hypothesize that *Vegf* expression is regulated by C/EBPβ in GCs during ovulation.

Not only transcription factors, but also epigenetic mechanisms regulate gene expression^14–16^. Histone modification is one of them and affects the chromatin structure, which regulates the accessibility of transcription factors into gene promoter or enhancer region^17–19^. In previous studies of GCs in rats undergoing luteinization^13,20,21^, we reported that changes of histone modifications and chromatin remodeling are involved in the regulation of three luteinization-related genes: steroidogenic acute regulatory protein (*StAR*), aromatase (*Cyp19a1*) and cytochrome P450 cholesterol side-chain cleavage enzyme (*Cyp11a1*). These findings raised the possibility that *Vegf* expression is regulated not only by transcription factors but also by an epigenetic mechanism.

In this study, we showed that C/EBPβ, but not HIF1α, regulates *Vegf* expression in rat GCs undergoing luteinization.

## Results

### *Vegf* mRNA expression

**Figure 1:**
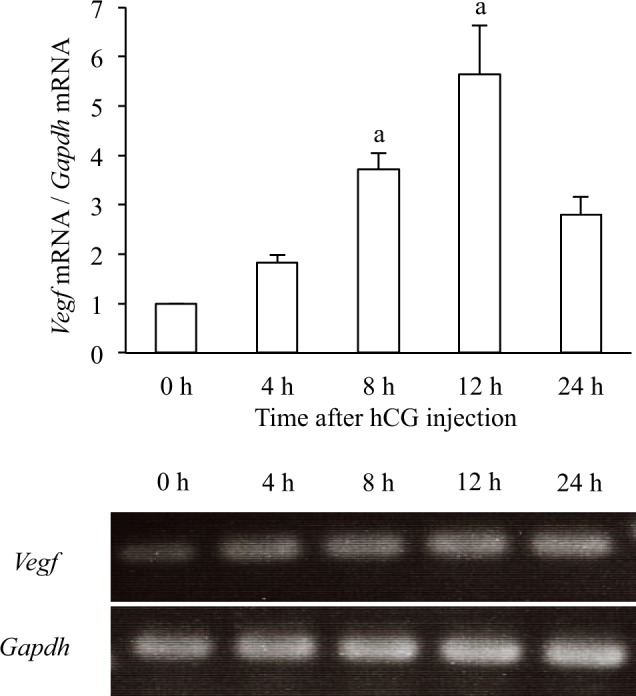
*Vegf* mRNA expression in rat GCs undergoing luteinization. The relative mRNA levels of *Vegf* in GCs before (0 h) and 4, 8, 12, 24 h after hCG injection were assessed by qPCR. *Gapdh* was used as an internal control. Data were shown as a ratio of those of 0 h. Each value represents the mean ± SEM of 6 animals. a, P < 0.05 vs. 0 h. mRNA expression was also assessed by RT-PCR. The images of representative gels are shown.

Human chorionic gonadotropin (hCG) was injected to rats to induce luteinization. GCs were isolated from ovarian follicles. *Vegf* mRNA levels in the GCs gradually increased and reached a peak at 12 h after hCG injection (Fig. 1).

### C/EBPβ and HIF1α protein expressions

**Figure 2:**
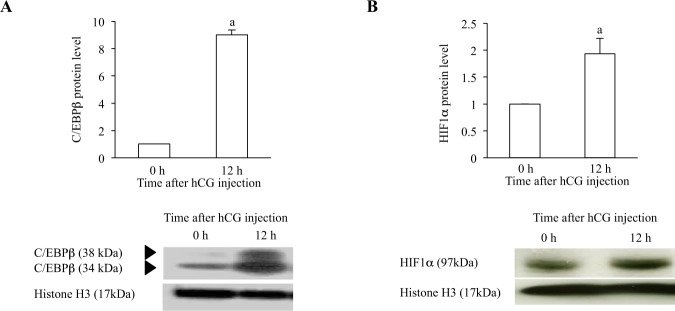
C/EBPβ and HIF1α protein expressions in rat GCs undergoing luteinization. Whole cell lysate were isolated from the GCs (0 h and 12 h). Protein levels of C/EBPβ (**A**) and HIF1α (**B**) were examined by Western blotting. Protein levels of histone H3 were also assessed to ensure equal amount of proteins. Western blotting was repeated 3 times, and the representative immunoblots are shown. Quantification of bands were performed by using ImageJ and normalized with histone H3 levels. Data were shown as a ratio of those of 0 h. Each value represents the mean ± SEM of 3 independent experiments. a, P < 0.05 vs. 0 h.

We examined by Western blot analyses whether C/EBPβ and HIF1α protein expression levels were increased in rat GCs after hCG injection. C/EBPβ protein (Fig. 2A) and HIF1α protein (Fig. 2B) were expressed in rat GCs before luteinization (0 h) and were up-regulated by hCG injection (12 h).

### Binding activity of C/EBPβ and HIF1α to the *Vegf* promoter

**Figure 3:**
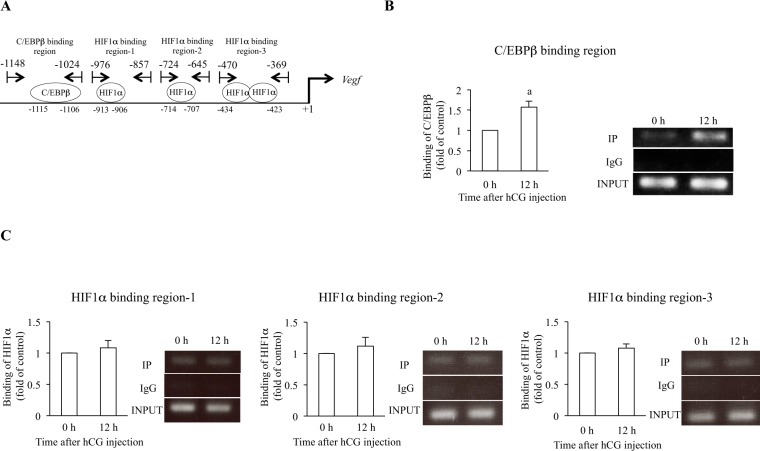
Binding activities of C/EBPβ and HIF1α to the *Vegf* promoter region in rat GCs undergoing luteinization. (**A**) Location of the binding sites for transcription factors. Primers for ChIP assay were designed to surround the C/EBPβ binding site (−1148 bp to −1024 bp) and the HIF1α binding sites (−976 bp to −857 bp; region-1, −724 bp to −645 bp; region-2, −470 bp to −369 bp; region-3). Binding activities of C/EBPβ (**B**) and HIF1α (**C**) before (0 h) and 12 h after hCG injection were analyzed by ChIP assay. GCs from three rats were pooled at each time point to use for ChIP assay. ChIP assay was performed with antibodies to C/EBPβ, HIF1α and control IgG. The relative binding activities of C/EBPβ and HIF1α were assessed by qPCR. The ratio of IP DNA to INPUT DNA sample was calculated. Data were shown as a ratio of those of 0 h. Each value represents the mean ± SEM of 3 independent experiments. a, P < 0.05 vs. 0 h. PCR products from IP DNA and INPUT DNA were also electrophoresed. The images of representative gels are shown.

The JASPAR database lists consensus binding sequences for several transcription factors in the *Vegf* promoter, including C/EBPβ (−1115 bp to −1106 bp) and HIF1α (−913 bp to −906, −714 bp to −707 bp, −434 bp to −423 bp) (Fig. 3A). Because C/EBPβ and HIF1α protein levels were increased after hCG injection in rat GCs (Fig. 2), we hypothesized that C/EBPβ and HIF1α bind to the *Vegf* promoter region and regulate *Vegf* expression. Therefore, we designed chromatin immunoprecipitation (ChIP) primers surrounding these sites, −1148 bp to −1024 bp (C/EBPβ binding region), −976 bp to −857 bp (HIF1α binding region-1), −724 bp to −645 bp (HIF1α binding region-2) and −470 bp to −369 bp (HIF1α binding region-3), respectively (Fig. 3A). The binding activity of C/EBPβ to the *Vegf* promoter region was significantly increased in GCs 12h after hCG injection (Fig. 3B). The binding activities of HIF1α were observed in all HIF1α binding regions before luteinization (Fig. 3C, 0 h). However, they were not increased 12 h after hCG injection (Fig. 3C, 12 h). These results indicate that C/EBPβ, but not HIF1α, regulates *Vegf* gene expression by binding to the promoter region in rat GCs undergoing luteinization.

### Effect of C/EBPβ knockdown on *VEGF* mRNA expression

**Figure 4:**
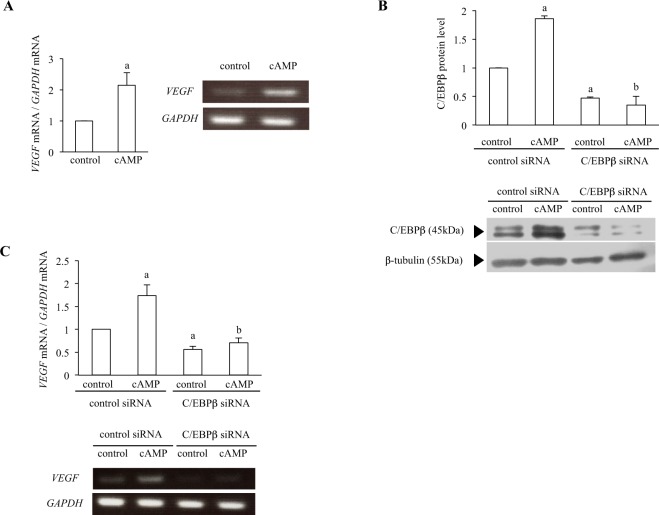
Effect of C/EBPβ knockdown on *VEFG* mRNA expression. (**A**) KGN cells were treated with or without cAMP (0.5 mM) for 24 h. The relative mRNA levels of *VEGF* were assessed by qPCR. *GAPDH* was used as an internal control. Data were shown as a ratio of the control treatment sample. Each value represents the mean ± SEM of 3 independent incubations. a, P < 0.05 vs. control sample. mRNA expression was also assessed by RT-PCR. The images of representative gels are shown. (**B**) KGN cells were transfected with a siRNA targeted against C/EBPβ or with a nontargeting siRNA as a control. After 24 h of transfection, cells were treated with or without cAMP for 24 h. Whole cell lysates were prepared and protein levels of C/EBPβ were examined by Western blotting to confirm the C/EBPβ knockdown. Protein levels of β−tubulin were also assessed to ensure equal amount of proteins. Western blotting was repeated 3 times, and the representative immunoblots are shown. Quantification of bands were performed by using ImageJ and normalized with β−tubulin levels. Data were shown as a ratio of the control treatment sample. Each value represents the mean ± SEM of 3 independent experiments. a, P < 0.05 vs. control treatment; b, P < 0.05 vs. cAMP treatment in the control siRNA. (**C**) The relative mRNA levels of *VEGF* were assessed by qPCR. *GAPDH* was used as an internal control. Data were shown as a ratio of the control treatment in the control siRNA. Each value represents the mean ± SEM of 3 independent incubations. a, P < 0.05 vs. control treatment in the control siRNA. b, P < 0.05 vs. cAMP treatment in the control siRNA. mRNA expression was also analyzed by RT-PCR. All PCR products were electrophoresed on the same gel and the ethidium bromide-stained gel is a representative of 3 independent incubations.

To elucidate the involvement of C/EBPβ in *Vegf* mRNA expression, we examined the effect of C/EBPβ knockdown on *Vegf* mRNA expression. Because the efficiency of knockdown in primary GCs is quite low, we used KGN cells (human granulosa tumor cells) that are widely used for experiments on luteinization^22^. Because KGN cells do not respond to hCG stimulation, cAMP, a second messenger of hCG, was used to induce luteinization in KGN cells as reported previously^22,23^. First, we confirmed that *VEGF* mRNA was increased by cAMP stimulation in KGN cells (Fig. 4A). Knockdown of C/EBPβ by siRNA decreased the protein expression levels of basal (control) and cAMP-increased C/EBPβ (Fig. 4B). The expression of cAMP-increased *VEGF* mRNA was suppressed by the knockdown of C/EBPβ (Fig. 4C). The basal *VEGF* mRNA expression in KGN cells without cAMP stimulation was also significantly decreased by the knockdown of C/EBPβ (Fig. 4C). These results suggest that C/EBPβ is involved in not only cAMP-induced *Vegf* expression but also basal expression of *Vegf*.

### Transcriptional activity

**Figure 5:**
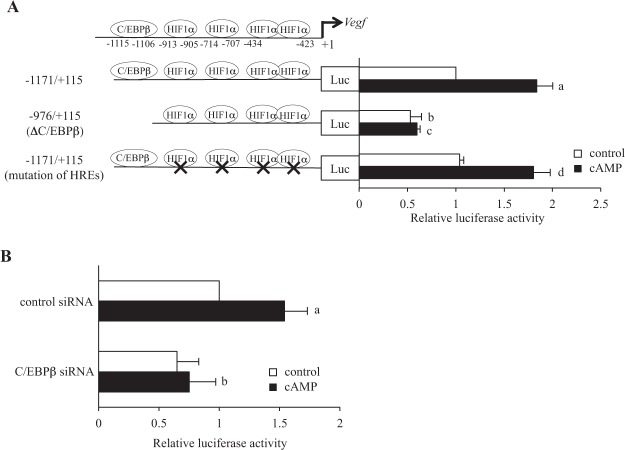
Transcriptional activities of the C/EBPβ binding site in rat *Vegf* promoter region. (**A**) The reporter constructs, −1171 bp to +115 bp, −976 bp to +115 bp (ΔC/EBPβ) and −1171 bp to +115 bp (mutation of HREs) were transfected into KGN cells. After 24 h of transfection, cells were treated with and without cAMP for 24 h. The firefly luciferase activity was normalized according to *Renilla* luciferase activities. Values of the luciferase activities were expressed as a ratio of control treatment with −1171 bp to +115 bp. Each value represents the mean ± SEM of 3 independent incubations. a, P < 0.05 vs. control treatment of the construct of −1171 bp to +115 bp. b, P < 0.05 vs. control treatment of the construct of −1171 bp to +115 bp. c, P < 0.05 vs. cAMP treatment of the construct of −1171 bp to +115 bp. d, P < 0.05 vs. control treatment of the construct of −1171 bp to +115 bp (mutation of HREs). (**B**) The reporter construct of −1171 bp to +115 bp and siRNA (C/EBPβ siRNA or non-targeting siRNA) were transfected into KGN cells. After 24 h of transfection, cells were treated with and without cAMP for 24 h. The firefly luciferase activity was normalized according to *Renilla* luciferase activities. Values of the luciferase activities were expressed as a ratio of control treatment with −1171 bp to +115 bp. Each value represents the mean ± SEM of 3 independent incubations. a, P < 0.05 vs. control treatment. b, P < 0.05 vs. cAMP treatment in the control siRNA.

The transcriptional activity of the C/EBPβ binding site in the rat *Vegf* promoter region was measured by a luciferase assay using KGN cells transfected with different rat *Vegf* promoter constructs (Fig. 5A). When cells were transfected with the promoter construct (−1171 bp to +115 bp), cAMP significantly increased the luciferase activities. Deletion of the C/EBPβ binding site completely blocked it (the construct of −976 bp to +115 bp) and the cAMP-induced luciferase activities were also decreased to the basal level (without cAMP stimulation). Mutation of the HIF1α binding sites (the mutated construct of −1171 bp to +115 bp) did not affect either cAMP-increased or basal luciferase activities. We further examined the luciferase activities of the rat *Vegf* promoter region under the condition of C/EBPβ knockdown. Both the construct of −1171 bp to +115 bp and siRNA were transfected to KGN cells and then cells were treated with cAMP. As shown in Fig. 5B, the increase in luciferase activities by the stimulation of cAMP was completely blocked by the knockdown of C/EBPβ. We also examined the effect of CoCl_2_, which induces HIF1α protein expression^24^, on the luciferase activities. CoCl_2_ increased the luciferase activities of the *Vegf* promoter region, and mutation of HIF1α binding sites blocked it (Supplementary Figure 1). These findings show that HIF1α binding sites in the rat *Vegf* promoter region have the transcriptional activities under CoCl_2_ stimulation, but not under cAMP stimulation.

### Histone modifications, chromatin structure, and binding activities of enhancer of zeste homolog 2 (EZH2)

**Figure 6:**
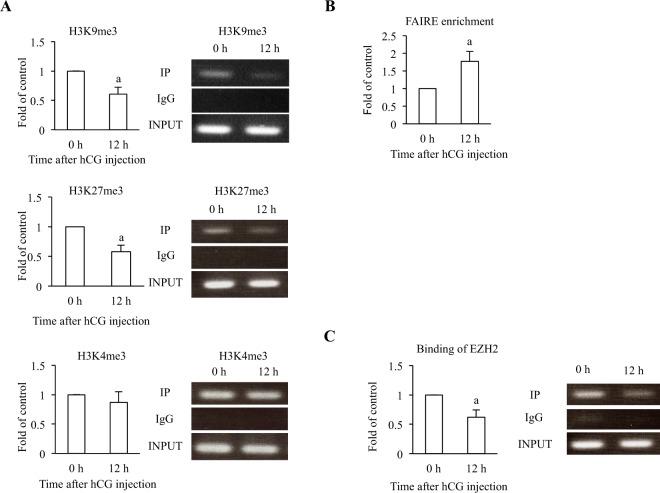
Histone modifications, chromatin structure and binding activity of EZH2 to the C/EBPβ binding region in the *Vegf* promoter in rat GCs undergoing luteinization. GCs were obtained from rats treated with eCG before (0 h) and 12 h after hCG injection. GCs from three rats were pooled at each time point to use for ChIP assay and FAIRE-qPCR. (**A**) The levels of H3K4me3, H3K9me3 and H3K27me3 of the C/EBPβ binding region were analyzed by ChIP assay. ChIP assay was performed with antibodies to H3K4me3, H3K9me3, H3K27me and control IgG. The relative levels of H3K4me3, H3K9me3 and H3K27me3 were analyzed by real-time PCR. The ratio of IP DNA to INPUT DNA sample was calculated. Data were shown as a ratio of those of 0 h. Each value represents the mean ± SEM of 3 independent experiments. a, P < 0.05 vs. 0 h. All PCR products from IP DNA or INPUT DNA were electrophoresed on the same gel and the representative ethidium bromide-stained gels are shown at the right. (**B**) To determine the changes of the chromatin structure in the C/EBPβ binding region, a FAIRE-qPCR was performed with same primers used in ChIP assay. The ratio of FAIRE enrichment in the *Vegf* promoter region was calculated. Data were shown as a ratio of those of 0 h. Each value represents the mean ± SEM of 4 independent experiments. a, P < 0.05 vs. 0 h. (**C**) Binding activity of EZH2 to the C/EBPβ binding region was analyzed by ChIP assay. ChIP assay was performed with antibodies to EZH2 and control IgG. The relative levels of EZH2 binding activities were analyzed by real-time PCR. The ratio of IP DNA to INPUT DNA sample was calculated. Data were shown as a ratio of those of 0 h. Each value represents the mean ± SEM of 3 independent experiments. a, P < 0.05 vs. 0 h. PCR products from IP DNA and INPUT DNA were also electrophoresed. The images of representative gels are shown.

Because not only transcription factors but also epigenetic mechanisms regulate gene expression^14–16^, we examined in rat GCs whether hCG stimulation changes histone modification levels of the C/EBPβ binding region in the *Vegf* promoter. The levels of H3K9me3 and H3K27me3, which are correlated with the suppression of transcription, significantly decreased at 12 h after hCG injection. The level of H3K4me3, which is correlated with the activation of transcription, was not affected by hCG injection (Fig. 6A).

The chromatin remodeling of the C/EBPβ binding region after hCG injection were examined by FAIRE (formaldehyde-assisted isolation of regulatory elements)-qPCR. The relative FAIRE enrichment ratio in this region increased by hCG injection (Fig. 6B), indicating that hCG stimulation loosens the chromatin structure of the C/EBPβ binding region.

We examined whether hCG stimulation changes the binding of EZH2, which induces H3K27me3^25,26^, to the C/EBPβ binding region. The EZH2 binding activity to the C/EBPβ binding region, as measured by ChIP assay, significantly decreased after hCG injection (Fig. 6C).

## Discussion

The present study demonstrated that C/EBPβ is an important transcription factor regulating *Vegf* expression in rat GCs undergoing luteinization during ovulation. C/EBPβ is well known to be transcription factor necessary for ovulation^12^, and belongs to the ERK-1/2 signaling pathway, which activates in GCs undergoing luteinization. We reported that C/EBPβ up-regulates the expression of *StAR* and *Cyp11a1* and promotes the production of progesterone, which play central roles in follicle rupture and subsequent CL formation^13,20^. Our results show a new finding that *Vegf* is one of downstream target genes of C/EBPβ in rat GCs after the LH surge, suggesting that C/EBPβ contributes to angiogenesis for CL formation by up-regulating *Vegf* expression.

This is the first report to show the involvement of C/EBPβ in the regulation of *Vegf* gene expression in rat GCs. The promoter region of the rat *Veg*f gene has the consensus binding sequence of C/EBPβ, which is highly conserved among species^27^. However, this site has not been examined so far. C/EBPβ clearly binds to this site in rat GCs undergoing luteinization (Fig. 3B). In addition, by knockingdown C/EBPβ protein in KGN cells, we demonstrated that C/EBPβ regulates the basal and cAMP-increased *Vegf* mRNA expressions (Fig. 4C). Furthermore, the C/EBPβ binding site has transcriptional activity (Fig. 5). Taken together, these findings show that C/EBPβ up-regulates *Vegf* gene expression through the binding to the novel regulatory region in rat *Vegf* promoter region in GCs undergoing luteinization.

HIF1α has been reported to regulate *Vegf* gene expression by binding to the promoter region in many types of cells^28,29^. HIF1α protein is expressed in granulosa cells before luteinization^30,31^ and is up-regulated by hCG stimulation^10,32–34^, which is consistent with our findings (Fig. 2B). Because both VEGF and HIF1α expression show the same trend toward luteinization, HIF1α was thought to be involved in the increase of *Vegf* expression by luteinization. However, the direct evidences showing that the increase in *Vegf* expression by luteinization is mediated by HIF1α have not been reported so far. We proved by ChIP assay that the binding activities of HIF1α were observed in all HIF1α binding regions in rat *Vegf* promoter region before luteinization (Fig. 3C, 0 h), but these binding activities were not increased after hCG injection (Fig. 3C, 12 h). This result suggests that HIF1α does not contribute to the up-regulation of *Vegf* expression by hCG stimulation. In addition, we showed by luciferase assay that hypoxia responsible elements (HREs) in rat *Vegf* promoter region are not involved in the regulation of *Vegf* expression by cAMP. In this study, we did not show the involvement of HIF1α in the regulation of *Vegf* in our experimental condition including the use of KGN cells. However, we do not exclude the role of HIF1α in *Vegf* induction during luteinization, because HIF1α may regulate *Vegf* expression at different time points of luteinization process, or bind to an unknown promoter or enhancer region of *Vegf* gene undergoing luteinization in GCs. On the other hand, Rico *et al*.^35^ showed that HRE deficient mice responded to LH with an increase in *Vegf* expression in granulosa cells *in vivo*, suggesting that *Vegf* expression by LH is not regulated by HIF1α in mouse granulosa cells, which supports our findings. Kim *et al*.^10^ used Echinomycin, which inhibits the binding ability of HIF1α to HREs, and found that Echinomycin inhibits hCG-increased *Vegf* mRNA expression in mice granulosa cells. Although they concluded that hCG up-regulates *Vegf* expression through HIF1α, it should be noted that Echinomycin directly affects many gene expressions. Taken together, we concluded that C/EBPβ is an important factor to regulate *Vegf* gene expression in rat GCs undergoing luteinization after the LH surge.

Although there are several reports showing that HIF1α is associated with *Vegf* mRNA expression in GCs, most of them are based on the data from *in vitro* experiments done under the conditions of hypoxia or CoCl_2_ stimulation. Martinez-Chequer *et al*.^36^ reported that CoCl_2_ increased *VEGF* expression in monkey GCs. Alam *et al*.^37^ reported that CoCl_2_ induced HIF1α protein expression in rat GCs. Kim *et al*.^10^ showed that HIF1α is involved in *Vegf* expression induced by CoCl_2_ in mouse GCs. Yalu *et al*.^24^ showed the involvement of HIF1α in the CoCl_2_-induced *VEGF* expression by knockingdown HIF1α in human GCs. Our results also showed that HIF1α contributes to *Vegf* expression under CoCl_2_ stimulation, but not under cAMP stimulation (Supplementary Figure 1). From these results, it is likely that HIF1α is involved in the regulation of *Vegf* expression in GCs under CoCl_2_ stimulation *in vitro*.

Our data also showed that epigenetic changes such as histone modifications and chromatin remodeling occurred in the *Vegf* promoter region in luteinizing rat GCs. These changes included decreases of H3K9me3 and H3K27me3 levels along with a decrease of EZH2 binding to the C/EBPβ binding region in the *Vegf* promoter all of which are associated with the activation of transcription by loosening the chromatin^38^. This result is consistent with our previous findings that stimulating rats with hCG caused decreases in H3K9me3, H3K27me3 and EZH2 binding of the *Cyp11a1* promoter region in GCs and increases in their mRNA expressions^20^. Our results showed that hCG loosened the chromatin structure of the *Vegf* promoter region, allowing C/EBPβ to access its response element of the *Vegf* promoter region. These findings suggest that these epigenetic changes are closely associated with the up-regulation of *Vegf* expression in GCs undergoing luteinization. H3K4me3 is a histone modification related to active transcription^39,40^. In our study, the levels of H3K4me3 were not changed by hCG stimulation. This is not surprising, because the changes of H3K4me3 levels are not always correlated with those of other histone modifications^18^, and because gene expression is regulated by not only one histone modification, but also the combination of various histone modifications^13^.

In summary, the present study shows a molecular mechanism by which *Vegf* is up-regulated in rat GCs undergoing luteinization after the LH surge. We found that C/EBPβ, but not HIF1α, regulates *Vegf* gene expression by binding to the novel binding site in the rat *Vegf* promoter region. In addition to transcription factors, histone modifications and chromatin structure of the *Vegf* promoter region are involved in the regulation of *Vegf* expression. Because *Vegf* plays a key role in angiogenesis, our results should help to better understand the regulation of angiogenesis in GCs undergoing luteinization during ovulation.

## Methods

This study was reviewed and approved by The Committee for the Ethics on Animal Experiment in Yamaguchi University Graduate School of Medicine. All experiments were performed in accordance with relevant guidelines and regulations.

### Animal models

Female Sprague Dawley rats (21- to 24-day old) were purchased from Japan SLC (Hamamatsu, Japan). They were injected subcutaneously with 15 IU of equine chorionic gonadotropin (eCG) (Sigma, St. Louis, MO, USA) to promote follicular growth followed by 15 IU of hCG (Sigma) injection to induce ovulation and luteinization. The ovaries were obtained before hCG (0), and 4, 8, 12, and 24 hours (h) after hCG injection. The follicles were punctured to isolate GCs. Cells were centrifuged and washed in PBS and provided for the experiments.

### Cell culture

KGN cells (Nikon, Tokyo, Japan), which were derived from a human granulosa cell tumor, were cultured in DMEM/Ham’s F-12 medium.

### Quantitative real-time PCR

RNA was extracted with RNeasy Mini (QIAGEN, Chatsworth, CA, USA) and was converted to cDNA with reverse transcriptase (Invitrogen, Waltham, MA, USA) as reported previously^41^. Quantitative real time PCR reaction was carried out as described previously^42,43^. Primer sequences are listed in Table 1.

**Table 1:** Primers Used in This Study.

Gene	Primer (5′ to 3′)	Amplification Size (bp)
RT-PCR		
*Vegf* (rat)	For, CACTGGACCCTGGCTTTACT Rev, GACGTCCATGAACTTCACCA	111
*Hif1α* (rat)	For, TGGTGCTAACAGATGATGGTG Rev, CATGGTCACATGGATGGGTA	123
*Gapdh* (rat)	For, CTCATGACCACAGTCCATGC Rev, TTCAGCTCTGGGATGACCTT	155
*VEGF* (human)	For, CCTTGCTGCTCTACCTCCAC Rev, GCAGTAGCTGCGCTGATAGA	119
*GAPDH* (human)	For, AGGTGAAGGTCGGAGTCA Rev, GGTCATTGATGGCAACAA	99
ChIP-qPCR		
*Vegf* promoter of C/EBPβ binding region	For, ATCCTACCCGGAGTTGGTG Rev, ACTAAGGCCAGTGTGCCAAT	125
*Vegf* promoter of HIF1α binding region -1	For, GAACAAGGGCTTCTGTCTGC Rev, GGAAGCCGAGCAGTTAGTCA	120
*Vegf* promoter of HIF1α binding region -2	For, TACTTGCCTTCCACGTAGCC Rev, CCCAACAGTTGCTTGTTTGA	80
*Vegf* promoter of HIF1α binding region -3	For, GAGTCTGCGTGAGGAAGGAC Rev, CCTGGTCTTCTCCCCTACCT	102
Luciferase assay		
−1171/+115	For, GGTACCGAGGGAGCCTTACCTCTACTCC Rev, AAGCTTGGCTGATGAGTCCGTTGAAT	1286
−976/+115 (ΔC/EBPβ)	For, GGTACCGAACAAGGGCTTCTGTCTGC Rev, AAGCTTGGCTGATGAGTCCGTTGAAT	1091
−1171/+115 (Mutation of HREs)-1	For, GCATACTCTGGCTTCCACAGGTCGTCTCCCTCCG Rev, GAAGCCAGAGTATGCACTGTGGAGTCTGGCAGAG	1286
−1171/+115 (Mutation of HREs) -2	For, GCCTTCAGAGTAGCCCCCCGCCCCATA Rev, GGCTACTCTGAAGGCAAGTATGCTTAT	1286
−1171/+115 (Mutation of HREs)-3,4	For, GTGCTCTCTCTCATGTGCGTGTGTGTCTGGGTATAGTGTG Rev, CATGAGAGAGAGCACTCACATTGACACACGCGTCCTTCC	1286

### Western blotting

Total protein were extracted and subjected to SDS-PAGE and then transferred to membrane as described previously^18,44^. The membranes were incubated for overnight with the following antibodies: C/EBPβ (Santa Cruz Biotechnology), HIF1α (Novus Biologicals, USA) and Histone H3 (Cell Signaling Technology, Tokyo, Japan). Amersham ECL Western Blotting Detection Reagent and hyperfilm-ECL (GE health care UK Ltd., Buckinghamshire, UK) were used to detect immunoblot bands.

### ChIP assay

The histone modification levels and recruitments of transcription factors were examined by ChIP assays as described previously^45^ with some modifications. The following antibodies were used in this assay: H3K4me3 (Upstate Biotechnology, Lake placid, NY, USA), H3K9me3 (Abcam, Cambridge, UK, USA), H3K27me3 (generous gift from Dr. Kimura), EZH2 (Cell Signaling Technology), C/EBPβ, HIF1α and non-specific normal IgG (Cell Signaling Technology). Real-time qPCR was performed with primers listed in Table 1 to calculate the relative enrichment.

### Transfection of siRNA

Transfection of siRNAs was performed as reported previously^46^. After 24 h of transfection, KGN cells were incubated with or without dibutyryl-cAMP (cAMP, 0.5 mM) (Sigma) for 24 h.

### Luciferase assay

Three 5′-flanking regions of the rat *Vegf* gene; 1) the construct including both C/EBPβ binding site and HIF1α binding sites (−1171 bp to +115 bp), 2) the construct lacking the C/EBPβ binding site (−976 bp to +115 bp) and 3) the construct that four HIF1α binding sites are mutated (−1171 bp to +115 bp), were amplified using the primer sets in Table 1. These constructs were inserted to pGL3 basic vector (Promega, Tokyo, Japan). Transfection of the constructs were performed as described^20^ and then cells were incubated with or without cAMP (0.5 mM) or CoCl_2_ (0.5 mM) for 24 h. Luciferase activities were examined as described^20^. Luciferase assays combined with C/EBPβ knockdown were performed as reported previously^23^. The reporter construct (the construct of −1171 bp to 115 bp) and siRNA (C/EBPβ siRNA or non-targeting siRNA) were transfected simultaneously. Then, KGN cells were incubated with or without cAMP and luciferase activities were examined. Luciferase assays were done in triplicate and repeated three times.

### FAIRE-qPCR

To examine the chromatin accessibility of C/EBPβ binding region in the *Vegf* promoter, FAIRE-qPCR was performed as described^47,48^ with some modifications. Real-time qPCR was performed to determine and calculate the FAIRE enrichment with same primers used in ChIP assay (Table 1).

### Statistical analyses

Unpaired student t-test was used to compare the mean values in two groups. To analyze differences between groups, one-way ANOVA followed by Tukey-Kramer test was used. P < 0.05 was considered significant.

## Electronic supplementary material

Supplementary information file

## References

[1] Fraser HM, Duncan WC (2009). SRB Reproduction, Fertility and Development Award Lecture 2008. Regulation and manipulation of angiogenesis in the ovary and endometrium. Reproduction, Fertility and Development.

[2] Sugino N, Matsuoka A, Taniguchi K, Tamura H (2008). Angiogenesis in the human corpus luteum. Reproductive Medicine and Biology.

[3] Kizuka F (2012). Involvement of bone marrow-derived vascular progenitor cells in neovascularization during formation of the corpus luteum in mice. Biol Reprod.

[4] Ferrara N (1998). Vascular endothelial growth factor is essential for corpus luteum angiogenesis. Nature medicine.

[5] Phillips HS, Hains J, Leung DW, Ferrara N (1990). Vascular endothelial growth factor is expressed in rat corpus luteum. Endocrinology.

[6] Ferrara N (2001). Role of vascular endothelial growth factor in regulation of physiological angiogenesis. American journal of physiology. Cell physiology.

[7] Berisha B, Schams D, Rodler D, Pfaffl MW (2016). Angiogenesis in The Ovary - The Most Important Regulatory Event for Follicle and Corpus Luteum Development and Function in Cow - An Overview. Anat Histol Embryol.

[8] Wissing ML (2014). Identification of new ovulation-related genes in humans by comparing the transcriptome of granulosa cells before and after ovulation triggering in the same controlled ovarian stimulation cycle. Human Reproduction.

[9] Watkins WM (2013). Hypoxia-induced expression of VEGF splice variants and protein in four retinal cell types. Exp Eye Res.

[10] Kim J, Bagchi IC, Bagchi MK (2009). Signaling by hypoxia-inducible factors is critical for ovulation in mice. Endocrinology.

[11] Loureiro RM, D’Amore PA (2005). Transcriptional regulation of vascular endothelial growth factor in cancer. Cytokine Growth Factor Rev.

[12] Fan HY, Liu Z, Johnson PF, Richards JS (2011). CCAAT/enhancer-binding proteins (C/EBP)-alpha and -beta are essential for ovulation, luteinization, and the expression of key target genes. Mol Endocrinol.

[13] Lee L (2013). Changes in histone modification and DNA methylation of the StAR and Cyp19a1 promoter regions in granulosa cells undergoing luteinization during ovulation in rats. Endocrinology.

[14] Maekawa R (2013). Genome-wide DNA methylation analysis reveals a potential mechanism for the pathogenesis and development of uterine leiomyomas. PLoS One.

[15] Sato S (2014). Potential mechanisms of aberrant DNA hypomethylation on the x chromosome in uterine leiomyomas. The Journal of reproduction and development.

[16] Tamura I (2014). Genome-wide analysis of histone modifications in human endometrial stromal cells. Mol Endocrinol.

[17] Dogan N (2015). Occupancy by key transcription factors is a more accurate predictor of enhancer activity than histone modifications or chromatin accessibility. Epigenetics Chromatin.

[18] Tamura I (2014). Importance of C/EBPbeta binding and histone acetylation status in the promoter regions for induction of IGFBP-1, PRL, and Mn-SOD by cAMP in human endometrial stromal cells. Endocrinology.

[19] Li B, Carey M, Workman JL (2007). The role of chromatin during transcription. Cell.

[20] Okada M (2016). Epigenetic Changes of the Cyp11a1 Promoter Region in Granulosa Cells Undergoing Luteinization During Ovulation in Female Rats. Endocrinology.

[21] Maekawa R (2016). Changes in gene expression of histone modification enzymes in rat granulosa cells undergoing luteinization during ovulation. J Ovarian Res.

[22] Nishi Y (2001). Establishment and characterization of a steroidogenic human granulosa-like tumor cell line, KGN, that expresses functional follicle-stimulating hormone receptor. Endocrinology.

[23] Mizutani T (2014). C/EBPbeta (CCAAT/enhancer-binding protein beta) mediates progesterone production through transcriptional regulation in co-operation with SF-1 (steroidogenic factor-1). The Biochemical journal.

[24] Yalu R, Oyesiji AE, Eisenberg I, Imbar T, Meidan R (2015). HIF1A-dependent increase in endothelin 2 levels in granulosa cells: role of hypoxia, LH/cAMP, and reactive oxygen species. Reproduction (Cambridge, England).

[25] Cao R (2002). Role of histone H3 lysine 27 methylation in Polycomb-group silencing. Science (New York, N.Y.).

[26] Czermin B (2002). Drosophila enhancer of Zeste/ESC complexes have a histone H3 methyltransferase activity that marks chromosomal Polycomb sites. Cell.

[27] Rosenbloom KR (2015). The UCSC Genome Browser database: 2015 update. Nucleic Acids Res.

[28] Goldberg MA, Schneider TJ (1994). Similarities between the oxygen-sensing mechanisms regulating the expression of vascular endothelial growth factor and erythropoietin. The Journal of biological chemistry.

[29] Forsythe JA (1996). Activation of vascular endothelial growth factor gene transcription by hypoxia-inducible factor 1. Mol Cell Biol.

[30] Boonyaprakob U, Gadsby JE, Hedgpeth V, Routh PA, Almond GW (2005). Expression and localization of hypoxia inducible factor-1alpha mRNA in the porcine ovary. Canadian journal of veterinary research = Revue canadienne de recherche veterinaire.

[31] Nishimura R, Okuda K (2010). Hypoxia is important for establishing vascularization during corpus luteum formation in cattle. The Journal of reproduction and development.

[32] van den Driesche S, Myers M, Gay E, Thong KJ, Duncan WC (2008). HCG up-regulates hypoxia inducible factor-1 alpha in luteinized granulosa cells: implications for the hormonal regulation of vascular endothelial growth factor A in the human corpus luteum. Mol Hum Reprod.

[33] Tam KK (2010). Hormonally regulated follicle differentiation and luteinization in the mouse is associated with hypoxia inducible factor activity. Mol Cell Endocrinol.

[34] Duncan WC, van den Driesche S, Fraser HM (2008). Inhibition of vascular endothelial growth factor in the primate ovary up-regulates hypoxia-inducible factor-1alpha in the follicle and corpus luteum. Endocrinology.

[35] Rico C (2014). HIF1 activity in granulosa cells is required for FSH-regulated Vegfa expression and follicle survival in mice. Biol Reprod.

[36] Martinez-Chequer JC, Stouffer RL, Hazzard TM, Patton PE, Molskness TA (2003). Insulin-like growth factors-1 and -2, but not hypoxia, synergize with gonadotropin hormone to promote vascular endothelial growth factor-A secretion by monkey granulosa cells from preovulatory follicles. Biol Reprod.

[37] Alam H (2009). Role of the phosphatidylinositol-3-kinase and extracellular regulated kinase pathways in the induction of hypoxia-inducible factor (HIF)-1 activity and the HIF-1 target vascular endothelial growth factor in ovarian granulosa cells in response to follicle-stimulating hormone. Endocrinology.

[38] Tamura I (2011). Differential effects of progesterone on COX-2 and Mn-SOD expressions are associated with histone acetylation status of the promoter region in human endometrial stromal cells. J Clin Endocrinol Metab.

[39] Rada-Iglesias A (2011). A unique chromatin signature uncovers early developmental enhancers in humans. Nature.

[40] Barski A (2007). High-resolution profiling of histone methylations in the human genome. Cell.

[41] Tamura I (2012). Induction of IGFBP-1 expression by cAMP is associated with histone acetylation status of the promoter region in human endometrial stromal cells. Endocrinology.

[42] Matsuoka A (2010). Progesterone increases manganese superoxide dismutase expression via a cAMP-dependent signaling mediated by noncanonical Wnt5a pathway in human endometrial stromal cells. J Clin Endocrinol Metab.

[43] Tamura I (2017). Novel Function of a Transcription Factor WT1 in Regulating Decidualization in Human Endometrial Stromal Cells and Its Molecular Mechanism. Endocrinology.

[44] Nishimoto Y (2018). Decreased carbonyl reductase 1 expression promotes tumor growth via epithelial mesenchymal transition in uterine cervical squamous cell carcinomas. Reprod Med Biol.

[45] Maekawa R (2016). Tissue-Specific Expression of Estrogen Receptor 1 Is Regulated by DNA Methylation in a T-DMR. Mol Endocrinol.

[46] Tamura I (2018). The distal upstream region of insulin-like growth factor-binding protein-1 enhances its expression in endometrial stromal cells during decidualization. The Journal of biological chemistry.

[47] Simon JM, Giresi PG, Davis IJ, Lieb JD (2012). Using formaldehyde-assisted isolation of regulatory elements (FAIRE) to isolate active regulatory DNA. Nat Protoc.

[48] Arase M (2017). Dynamics of chromatin accessibility during TGF-beta-induced EMT of Ras-transformed mammary gland epithelial cells. Sci Rep.

