# Supplementary material for: C/EBPβ regulates Vegf gene expression in granulosa cells undergoing luteinization during ovulation in female rats
Source: Sci Rep. 2019 Jan 24;9:714. doi: 10.1038/s41598-018-36566-y (PMC6345775; doi:10.1038/s41598-018-36566-y)
Supplement: Supplementary file 1 — Supplementary information file [file 41598_2018_36566_MOESM1_ESM.pdf]

Supplementary Information data

C/EBPβ regulates *Vegf* gene expression in granulosa cells undergoing luteinization during ovulation in female rats

Masahiro Shinagawa, Isao Tamura, Ryo Maekawa, Shun Sato, Yuichiro Shirafuta, Yumiko Mihara, Maki Okada, Toshiaki Taketani, Hiromi Asada, Hiroshi Tamura, Norihiro Sugino*

Department of Obstetrics and Gynecology, Yamaguchi University Graduate School of Medicine, Minamikogushi 1-1-1, Ube, 755-8505 Japan

*Corresponding author: Norihiro Sugino, M.D., Ph.D.

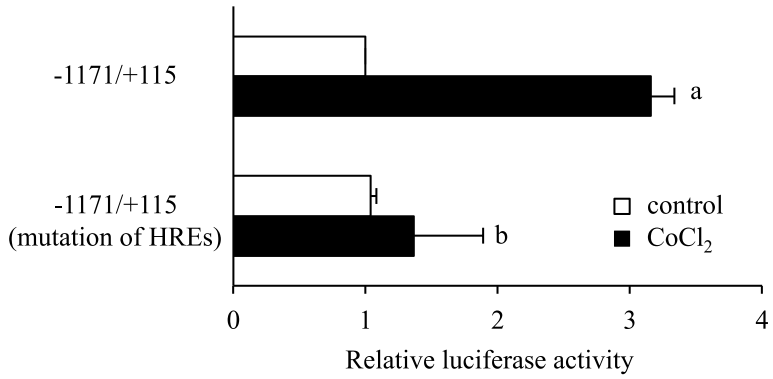

Supplementary Figure 1: Transcriptional activities of the C/EBP β binding site in rat Vegf promoter region under CoCl2 stimulation. The reporter constructs, -1171 bp to +115 bp and -1171 bp to +115 bp (mutation of HREs) were transfected into KGN cells. After 24 h of transfection, cells were treated with and without CoCl2 for 24 h. The firefly luciferase activity was normalized according to Renilla luciferase activities. Values of the luciferase activities were expressed as a ratio of control treatment with -1171 bp to +115 bp. Values are mean ± SEM of 3 different incubations. a, $P<0.05$ vs. control treatment of the construct of -1171 bp to +115 bp. b, $P<0.05$ vs. cAMP treatment of the construct of -1171 bp to +115 bp.

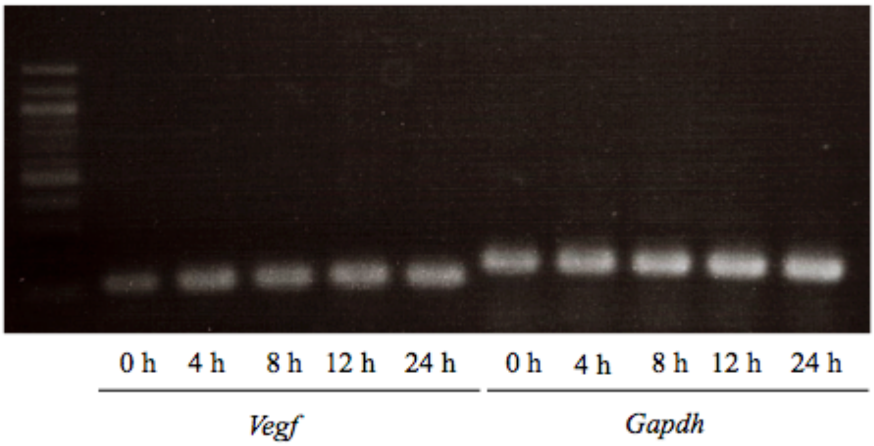

Supplementary Figure 2: *Vegf* mRNA expression in rat GCs undergoing luteinization. *Vegf* mRNA expression was analyzed by RT-PCR. *Gapdh* was used as an internal control. All PCR products were electrophoresed on the same gel and the representative ethidium bromide-stained gel is shown.

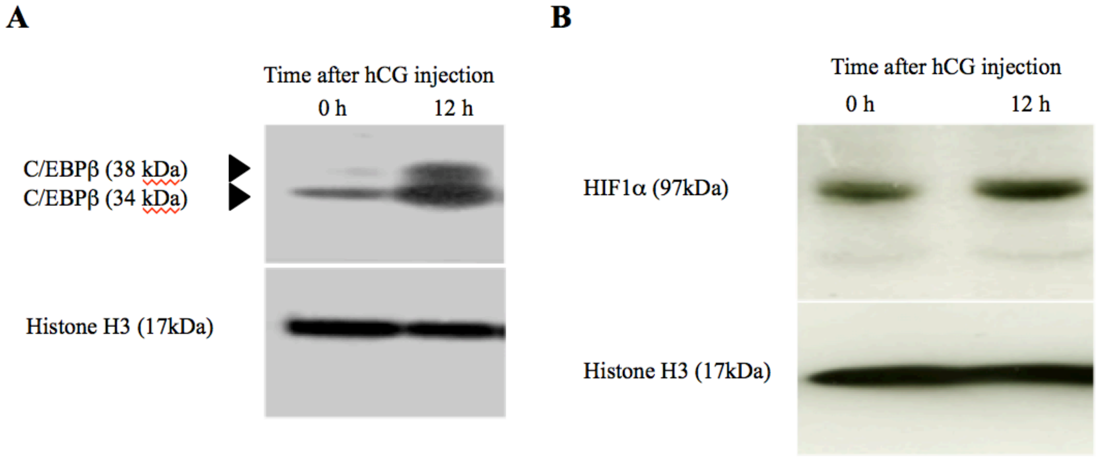

Supplementary Figure 3: HIF1α and C/EBPβ expressions in rat GCs undergoing luteinization. **A**, C/EBPβ protein levels were analyzed by Western blotting. Histone H3 was used as an internal control. **B**, HIF1α protein levels were analyzed by Western blotting. MES-SA cells cultured with 50 μM $CoCl_2$ were used as a positive control for HIF1α. Histone H3 was used as an internal control.

B

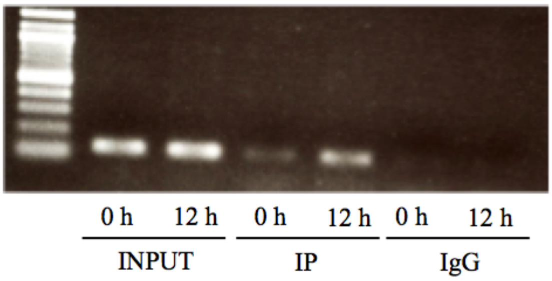

C

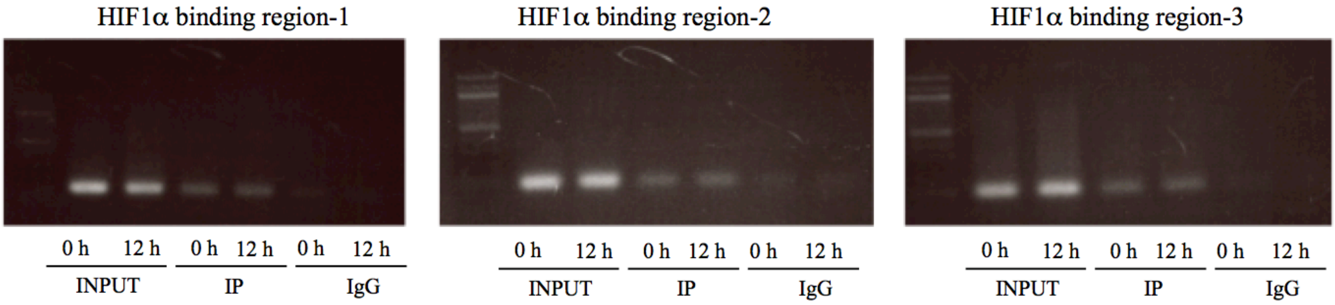

Supplementary Figure 4: Binding activities of C/EBPβ to the *Vegf* promoter region in rat GCs undergoing luteinization. **B,** Binding activities of C/EBPβ were analyzed by ChIP assay. All PCR products from INPUT or IP DNA were electrophoresed on the same gel and the representative ethidium bromide-stained gel is shown. **C,** Binding activities of HIF1α were analyzed by ChIP assay. All PCR products from INPUT or IP DNA were electrophoresed on the same gel and the representative ethidium bromide-stained gel is shown.

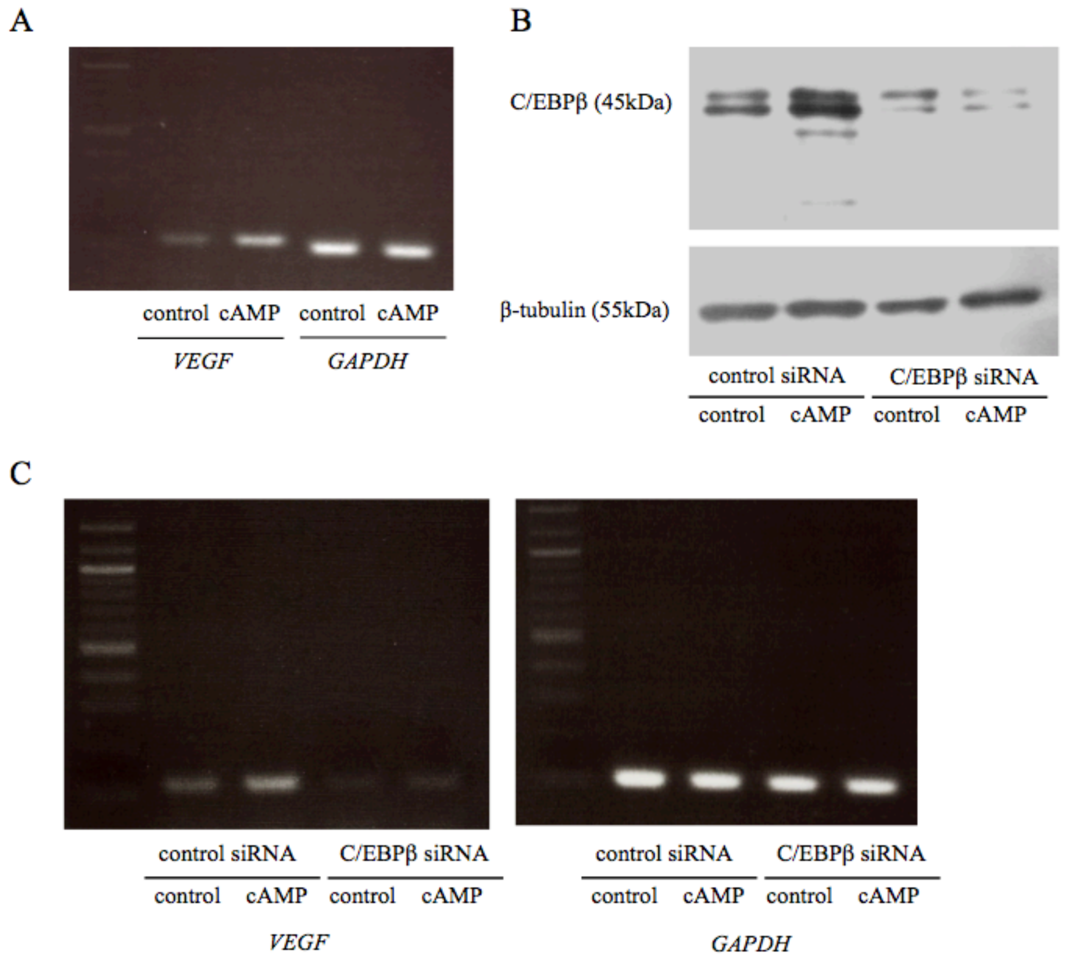

Supplementary Figure 5: Effect of C/EBPβ knockdown on *VEFG* mRNA expression. **A**, *VEGF* mRNA expression was analyzed by RT-PCR. *GAPDH* was used as an internal control. All PCR products were electrophoresed on the same gel and the representative ethidium bromide-stained gel is shown. **B**, KGN cells were transfected with a siRNA targeted against C/EBPβ or with a nontargeting siRNA as a control. 24 h after siRNA transfection, cells were treated with or without cAMP for 24 h. Whole cell lysates were prepared and subjected to Western blot to confirm the C/EBPβ knockdown. β-tubulin was used as an internal control. **C**, *VEGF* mRNA expression was analyzed by RT-PCR. *GAPDH* was used as an internal control. PCR products were electrophoresed on the same gel and the representative ethidium bromide-stained gels are shown.

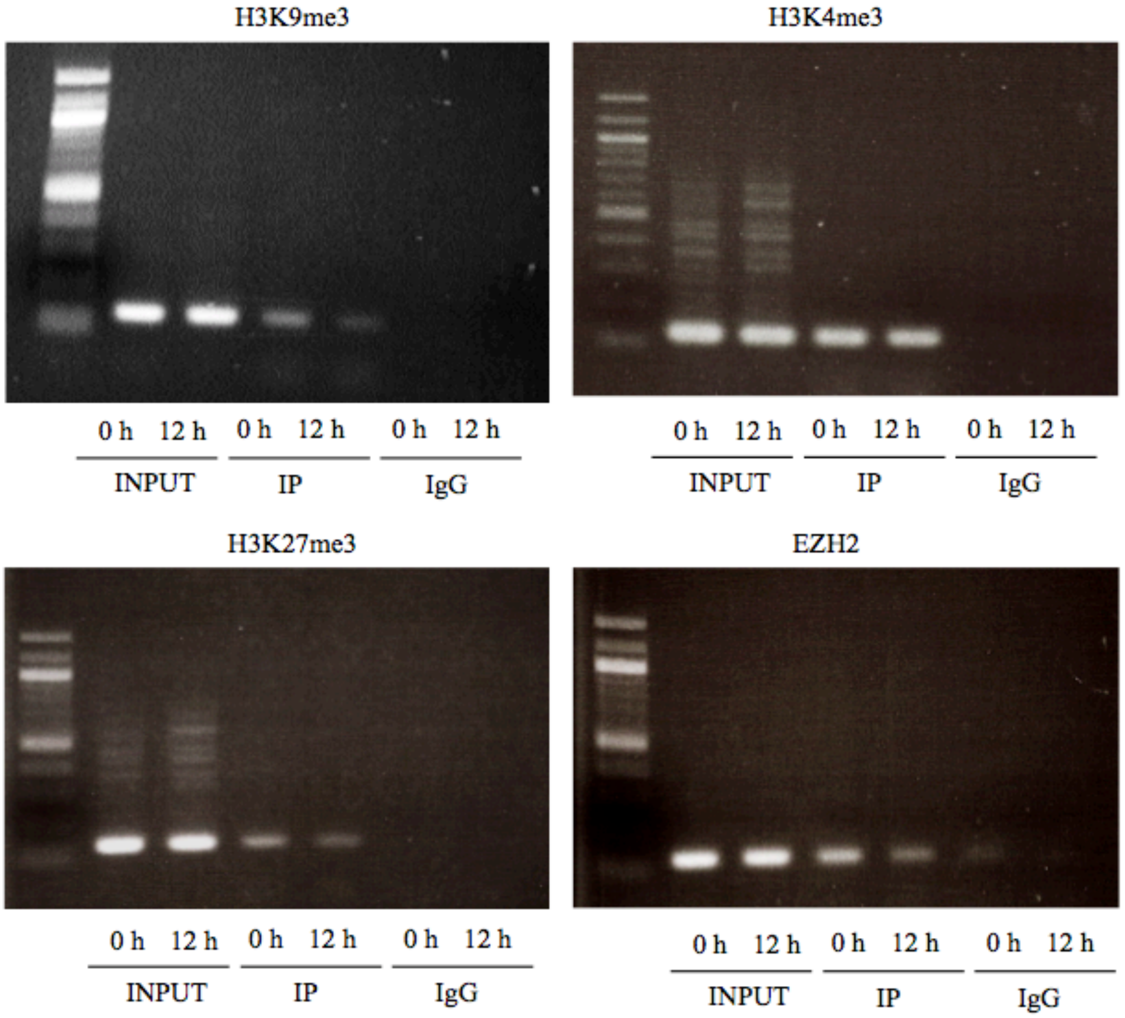

Supplementary Figure 6: Histone modifications and binding activity of EZH2 to the *Vegf* promoter region in rat GCs undergoing luteinization. The levels of H3K4me3, H3K9me3, H3K27me3 and Binding activity of EZH2 to the *Vegf* promoter region were analyzed by ChIP assay. Normal rabbit IgG was used as a negative control. All PCR products from INPUT or IP DNA were electrophoresed on the same gel and the representative ethidium bromide-stained gels are shown.

Supplementary Table 1. Antibody table

Peptide/protein target	Antigen sequence (if known)	Name of Antibody	Manufacturer, catalog #, and/or name of individual providing the antibody	Species raised in; monoclonal or polyclonal	Dilution used	RRID (required in revised MSs)
C/EBPβ		Anti-C/EBPβ antibody	Santa cruz:sc-150X	Rabbit; polyclonal	1/1000:WB, ChIP	AB_2260363
HIF1α		Anti-HIF1α antibody	Novus Biologicals: NB100-479	Rabbit; polyclonal	1/500: WB, 1/50: ChIP	AB_10000633
β-tubulin		Anti β-tubulin antibody	Sigma:T-4026	Mouse; monoclonal	1/1000: WB	AB_477577
Histone H3		Anti-Histone H3 antibody	Cell Signaling Technology: 4620S	Rabbit; monoclonal	1/4000: WB	AB_1904005
H3K4me3		Anti-H3K4me3 antibody	Upstate Biotechnology: 07-473	Rabbit; polyclonal	1/1000: ChIP	AB_1977252
H3K9me3		Anti-H3K9me3 antibody	Abcam, ab8898	Rabbit; polyclonal	1/1000: ChIP	AB_306848
H3K27me3		Anti-H3K27me3 antibody	Gift from Dr Hiroshi Kimura (Tokyo Kogyo University, Tokyo, Japan)	Rabbit; monoclonal	1/200: ChIP	
EZH2		Anti-EZH2 antibody	Cell Signaling Technology: 5246	Rabbit; monoclonal	1/100: ChIP	AB_10694683

Supplementary Table 1: Antibody table
